# Musketeer: a software tool for the analysis of titration data†

**DOI:** 10.1039/d4sc03354j

**Published:** 2024-08-29

**Authors:** Daniil O. Soloviev, Christopher A. Hunter

**Affiliations:** a Yusuf Hamied Department of Chemistry, University of Cambridge Lensfield Road Cambridge CB2 1EW UK herchelsmith.orgchem@ch.cam.ac.uk

## Abstract

Musketeer is a powerful open-source software tool for the analysis of titration data, featuring a simple cross-platform graphical interface for importing data directly from UV-vis, fluorescence and NMR spectrometers, or from spreadsheets. The fast data analysis algorithm can be used to obtain equilibrium constants for simple binding isotherms, as well as for more complicated systems with multiple competing equilibria. Applications of Musketeer for the analysis of a range of different supramolecular and biomolecular systems are illustrated, including titrations with multiple spectroscopically active species, competitive binding assays, denaturation experiments, optimisation of concentrations as variables. The software also includes a number of tools that can be used to select the binding isotherm that represents the best model to describe a dataset.

## Abstract 2

Musketeer is a versatile open-source software tool that can be used for the analysis of data from a range of different types of titration experiment, with no constraints on the spectroscopic technique or the complexity of the binding isotherm.

## Graphical abstract

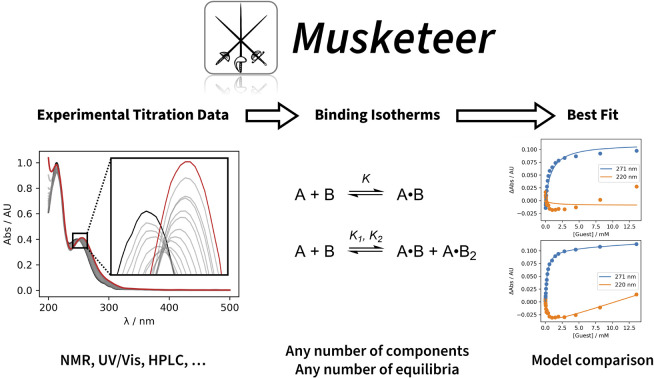

## 1 Introduction

Measuring association constants is a key activity in supramolecular chemistry, and the most common method used is a titration experiment.^1^ A property of the system that is sensitive to complexation, such as UV/vis absorbance or NMR chemical shift, is measured in solutions at varying concentrations, and the association constant is obtained by fitting the experimental data to a binding isotherm. Various tools for fitting titration data have been developed. Before the advent of nonlinear regression techniques, equilibrium constants were determined using linearisation techniques, such as the Benesi–Hildebrand plot.^2^ Modern computational methods use a variety of different software tools to fit more complicated binding isotherms.

Proprietary tools, such as HYPERQUAD and AFFINImeter, have seen widespread use, but there are limitations associated with cost, generality of applicability to instruments made by different manufacturers, and the range of different binding isotherms that can be analysed.^3–5^ Open-source software tools for analysing titration data, such as BindFit, SupraFit and SIVVU, have also been developed, both in the form of standalone software, or as plugins for commonly used proprietary programs, such as Mathematica and MATLAB, but these tools are often limited in the scope of experiments they support.^6–10^ A comprehensive review of the most popular tools *circa* 2012, and the strengths and limitations associated with each one, is provided by Thordarson.^11^

COGS and SPECFIT were among the first algorithms capable of handling arbitrarily complex speciation in the analysis of titration data for systems with multiple components and equilibria, but the original software is no longer supported.^12–14^ While some newer software tools implement the same speciation algorithms, these approaches rely on an iterative refinement of concentrations. As a result, the computational complexity scales poorly with the number of components in the system, and the functions are not guaranteed to be free from local minima. In addition, the implementation of complicated binding isotherms often requires the user to write their own code. Here we present a new open-source software tool for fitting titration data, Musketeer, which uses a new algorithm to rapidly calculate speciation for binding isotherms of arbitrary complexity. The software is controlled through an accessible cross-platform graphical interface that accepts data from any type of instrument or spectroscopic technique, provides a range of standard binding isotherms, and makes it straightforward for the user to implement more complicated models.

## 2 Approach

The aim of Musketeer is to support analysis of almost any kind of titration experiment without requiring researchers to manually derive the equations for complicated binding isotherms. Therefore, users only need to input the raw spectroscopic data, the concentrations of each component, and a chemical description of the model, *i.e.* specify the equilibria and spectroscopically active species. Musketeer then automatically converts the model to a set of equations that are solved to fit the data, returning optimised values for the variables and a set of publication-quality figures. There are three steps in setting up an analysis using Musketeer.

### 2.1. Experimental data

Firstly, a list of spectroscopic measurements must be provided for each addition. The spectra can contain any number of different signals, such as the NMR chemical shift of a single proton, or the UV/vis absorbance at hundreds of wavelengths. The data can either be entered directly into Musketeer through a built-in spreadsheet interface, imported from a CSV file, or automatically converted from a different file format using a Python function. By default, Musketeer can convert NMR data from a Mestrenova peak list or UV/vis absorption data from a Cary spectrophotometer, but users may supply their own functions to convert other file formats.

Next, the total concentration of each component present in solution for each addition is required. The concentrations can either be entered directly, or as stock solutions and addition volumes. Concentrations can also be entered as variables to be optimised using “?”. This function is important when working close to the tight-binding limit, where a very small error in concentration can have a very large effect on the quality of fit (see below). Optimisation of concentrations also allows for analysis of systems where not all of the concentrations are known. When dealing with multiple unknown variables, it can be beneficial to enter an initial guess for some variables, which can be done by entering a value prefixed with “∼”. The optimisation algorithm tends to converge quickly as long as initial guesses are within two orders of magnitude of the optimum value.

The last piece of data relating to the experiment is specification of whether the different species present in solution are in slow or fast exchange on the spectroscopic timescale.

### 2.2. Equilibria

Musketeer requires a description of the equilibria present in the system in order to calculate the concentrations of all species present after each addition. Some common binding isotherms can be selected directly from a dropdown menu, while the “Custom” option allows an arbitrary number of equilibria to be entered by specifying the stoichiometric composition of each species. In addition, polymeric species can be entered by specifying “*n*” as the stoichiometry. Polymers are described by two equilibrium constants, a dimerisation constant *K*_2_ and an elongation constant *K*_*n*_. These two equilibrium constants can be optimised as two different variables for cooperative polymerisation isotherms or constrained to be identical for an isodesmic polymerisation isotherm.

Relationships between different equilibrium constants are entered by selecting the “Custom” option in the dropdown menu. For example, if a host has two independent binding sites with identical binding affinities for the same guest, the global association constants for the 1 : 1 and 1 : 2 complexes, *K*_1_ and *K*_2_, can be derived from a single variable, *K*_micro_, which corresponds to the microscopic association constant for a single binding event (eqn (1)).1
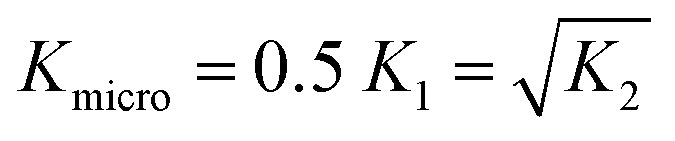


This case is sometimes referred to as the non-cooperative model,^6,8^ and the relationship is specified in Musketeer by using the “No cooperativity” option, which automatically sets up the non-cooperative model for any number of host binding sites. For more complicated relationships, the “Custom” option can be used to define any number of variables, such as microscopic binding constants, effective molarities, and statistical factors. Each global association constant is expressed as the corresponding statistical factor multiplied by the product of each variable raised to a specified exponent. There are additional options to constrain variables, such as equilibrium constants, to fixed values that have been measured in independent experiments.

### 2.3. Spectra

The relationship between the different species present in solution and the observed spectra must be specified. First, the spectroscopically active species are selected. In cases where different molecules contribute to different signals, such as in NMR spectra, “Custom, different per signal” is used to create lists of the species that contribute to each signal. It is possible to reduce the number of variables to be fitted, by specifying relationships between the spectra associated with different species. For example, one commonly used model is to assume that all binding events result in identical spectral changes, and this option can be selected from the dropdown menu. This model is sometimes referred to as the additive model. If used together with the “No cooperativity” option for the relationship between binding constants, it is known as the statistical model.^6,8^ There is a “Custom” option, which allows users to build more complicated relationships. If any spectra are known from prior experiments, these data can be entered directly to fix the values. There are additional options to constrain unknown spectra. For example, a “Nonnegative” option ensures that optimised UV/vis absorption spectra have positive absorbances at all wavelengths.

The use of the tools contained in Musketeer is illustrated below using experimental data from UV/vis absorption, fluorescence, NMR titrations, displacement assays and denaturation experiments. These examples show how Musketeer can be used to establish the most appropriate binding isotherm that is justified based on the experimental data. The denaturation example demonstrates how Musketeer can be used to build complicated models to describe multiple competing equilibria, but without introducing large numbers of variables that would lead to overfitting.

## 3 Multiple spectroscopically active species

In titration experiments under slow exchange conditions, the intensity of the observed signal at a specific wavelength is the sum of the contributions from all species present in solution. For example, if a UV/vis titration is used to study a 1 : 1 complex formed by a host H and a guest G, the total absorbance *A* at any given wavelength is given by eqn (2).

2 *A* = *ε*_H_[H] + *ε*_G_[G] + *ε*_H·G_[H·G]

where *ε*_*i*_ is the molar absorption coefficient of component *i*, [H] is the concentration of free host, [G] is the concentration of free guest, and [H·G] is the concentration of the 1 : 1 complex.

**Fig. 1:**
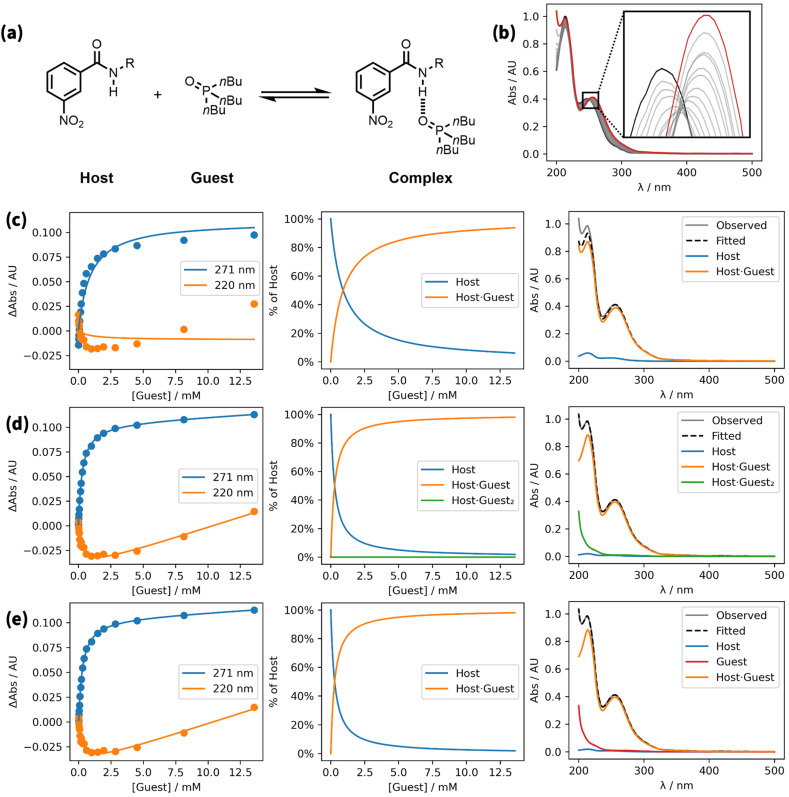
A UV/vis absorption titration (0.047 mM host in *n*-octane at 298 K).^16^ (a) Chemical structures. (b) Overlay of UV/vis absorption spectra. The initial spectrum is shown in black, the final spectrum in red. (c) Best fit to a 1 : 1 model allowing for absorption of the host and 1 : 1 complex only (*K*_1_ = 1120 M^−1^, RMSE = 7.8 × 10^−3^ AU). (d) Best fit to a 1 : 2 model allowing for absorption of the host, 1 : 1 complex, and 1 : 2 complex (*K*_1_ = 3800 M^−1^, *K*_2_ = 3.50 × 10^−8^ M^−2^, RMSE = 9.6 × 10^−4^ AU). (e) Best fit to a 1 : 1 model allowing for absorption of the host, free guest, and 1 : 1 complex (*K*_1_ = 3800 M^−1^, RMSE = 9.6 × 10^−4^ AU). Three plots are shown for each model: comparison of experimental absorbances at selected wavelengths (points) and the calculated values (lines), the calculated populations of species that contain host, and the calculated contributions to the endpoint spectrum (grey). R = 2-ethylhexyl.

To simplify the fitting process, the free guest is often assumed to be spectroscopically silent (*ε*_G_ = 0).^1,8,15^ However, even in cases where the guest has no obvious chromophore, accounting for the absorbance due to free guest can be important, because high concentrations of guest may be required to reach saturation in the binding isotherm. An example is shown in Fig. 1.^16^ A UV/vis absorption titration was carried out using an amide as the host and tri-*n*-butylphosphine oxide as the guest. Fig. 1c shows the fit of a 1 : 1 binding isotherm to the titration data, assuming that the guest does not absorb. Although this isotherm appears to provide a reasonable description of the blue data, the experimental points and the calculated line clearly diverge at high guest concentrations for the orange data. This result means that there is at least one other species that contributes to the observed UV/vis absorption spectra.

It is possible that there is a weak second binding event that leads to small amounts of a 1 : 2 complex at high guest concentrations. However, at high guest concentrations, the absorbance increased at all wavelengths, which suggests that absorption due to free guest may be the cause. Any number of spectroscopically active species can be considered in Musketeer, and Fig. 1d and e show the fits obtained by including either free guest or a 1 : 2 complex as the additional species. Both models provide a significantly better description of the Experimental data, reducing the root mean square error (RMSE) between the experimental and calculated spectra by an order of magnitude. It is worth noting that even though the simple 1 : 1 isotherm appears to provide a reasonable description of the blue data points in Fig. 1c, the association constant obtained from this fit (1120 M^−1^) deviates significantly from the value obtained in the other two models (3800 M^−1^).

## 4 Model evaluation

The example in Fig. 1 illustrates the result of fitting experimental data to three different models. Models with more optimisable variables always lead to a lower RMSE between the calculated and experimental spectra, so it is important to evaluate whether there is a sufficient improvement in the quality of the fit to justify the increase in the number of fitted variables required in more complicated models.^17^ Various mathematical techniques have been proposed for comparing multiple alternative binding isotherms based on a quality of fit parameter that is tensioned by the number of fitted variables: examples include the covariance of the fit, f-tests, Bayesian Information Criteria, and adjusted coefficients of determination.^1,8^ However, all of these approaches make assumptions that are not generally valid for titration data, namely that the total concentration of each molecule is known accurately, there are no errors in addition volume, there are no additional equilibria or impurities, errors are normally distributed random noise, and errors are identically distributed at each addition.^18–20^ The bootstrapping technique has been used to obtain error bounds on equilibrium constants without any assumption about the source of the errors,^10^ but further work would be required in order to develop this approach into a method that could be used for model selection. In the absence of a reliable mathematical technique for evaluating different models, Musketeer simply reports the RMSE between the calculated and experimental spectra, and the user should determine whether a more complicated model that uses more variables is justified based on the qualitative criteria outlined below.

### 4.1. Visual inspection of the raw data

Fig. 1b provides a good example. In a UV/vis titration, isosbestic points are usually observed for a simple two-state equilibrium, and in this case, the data can be fitted using a model with two spectroscopically active species. Close inspection of the magnified region in Fig. 1b shows that this titration does not have a well-defined isosbestic point, which means at least three spectroscopically active species must be considered in fitting the data. In an NMR titration, if a signal moves in one direction at the start of the titration and then moves in the other direction at the end of the titration, then a model with at least two complexes with different stoichiometries will be required to fit the data. As explained below, normalisation of the observed changes chemical shift for each signal is a useful technique for revealing the presence of more than one type of complex in an NMR titration.

### 4.2. Does the shape of the calculated curve match the experimental data points?

Fig. 1c provides a good example. The values of the experimental data shown in orange first decrease and then increase. The model in Fig. 1c is based on only two species, free and bound host, and so the calculated curve cannot change direction. This observation justifies the use of a more complicated model that includes an additional species.

### 4.3. Are the fitted parameters physically reasonable?

Fig. 1d provides a good example. The speciation plot shows that the population of the 1 : 2 complex is less than 1 part per million, because the association constant for formation the 1 : 2 complex is extremely low (*K*_2_ = 3.50 × 10^−8^ M^−2^). However, the calculated spectra show a substantial absorption for the 1 : 2 complex, which would require a molar absorption coefficient that is many orders of magnitude higher than the value for the free host or the 1 : 1 complex. This observation suggests that although the model achieves a good fit of the experimental data with a lower RMSE than the simple 1 : 1 model (9.6 × 10^−4^ AU compared with 7.8 × 10^−3^ AU), this result is an artefact of a very large calculated absorbance for a complex that is not formed to any extent in the experiment.

In contrast, the calculated spectra for the model that allows for guest absorption in Fig. 1e show that the increase in absorption observed for the orange data points comes from the tail of a very weak absorption at low wavelengths due to free guest, which is present at very high concentrations at the end of the titration. We conclude that the model used in Fig. 1e provides the best representation of this system. This example highlights the danger of overfitting by using too many variables. The model used in Fig. 1e produces a good fit with one additional variable (*ε*_G_). The model used in Fig. 1d has two additional variables (*K*_2_ and *ε*_H·G_2__), and the interplay of these variables produces a good fit, but only with values that are not physically reasonable.

### 4.4. Do the fitted parameters correspond to well-defined minima ?

**Fig. 2:**
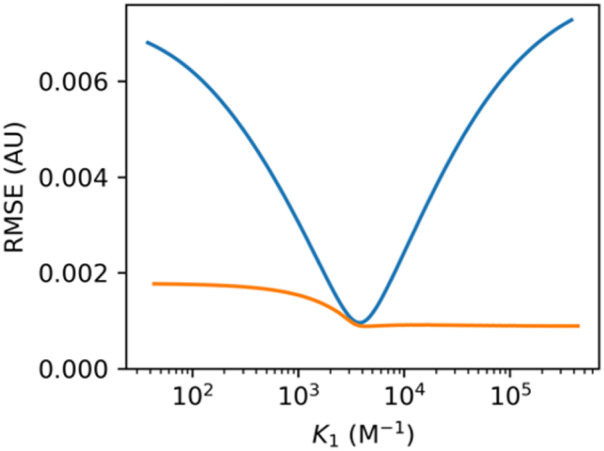
Relationship between the RMSE and the value of *K*_1_ for the UV/vis titration data in Fig. 1 fitted using a model allowing for absorption of the host, free guest, and 1 : 1 complex (blue) or a model allowing for absorption of the host, free guest, 1 : 1 complex and 1 : 2 complex (orange).

Models with too many optimisable variables lead to overfitting, because correlations between the parameters lead to multiple solutions with similar values of RMSE. This situation can be identified using the “RMSE plot” option in Musketeer, which iteratively fixes one of the parameters across a range of values, optimises the remaining variables, and plots the resulting relationship between the RMSE and the fixed parameter. For the example in Fig. 1 including the 1 : 2 complex in the model improved the fit shown in Fig. 1d and including guest absorption improved the fit shown in Fig. 1e, so one might be tempted to use a model that includes both guest absorption and the 1 : 2 complex. Fig. 2 shows the resulting relationship between the RMSE and the value of *K*_1_. The blue line shows the RMSE plot for *K*_1_ obtained using the model that includes the 1 : 1 complex and guest absorption only (*cf.*Fig. 1e). The orange line shows the RMSE plot for *K*_1_ obtained using the more complicated model that includes the 1 : 1 complex, the 1 : 2 complex and guest absorption. The minimum in RMSE is slightly lower for the more complicated model, as expected, but it is clear that the value of *K*_1_ cannot be reliably established using this model. The reason is that there are compensating changes in the other variables, such that any value of *K*_1_ greater than 3 × 10^3^ M^−1^ gives a similar RMSE. In contrast, there is a well-defined minimum in the RMSE plot for the simpler model, which shows that an optimal value of *K*_1_ can be accurately identified using this model.

### 4.5. Normalised plots

**Fig. 3:**
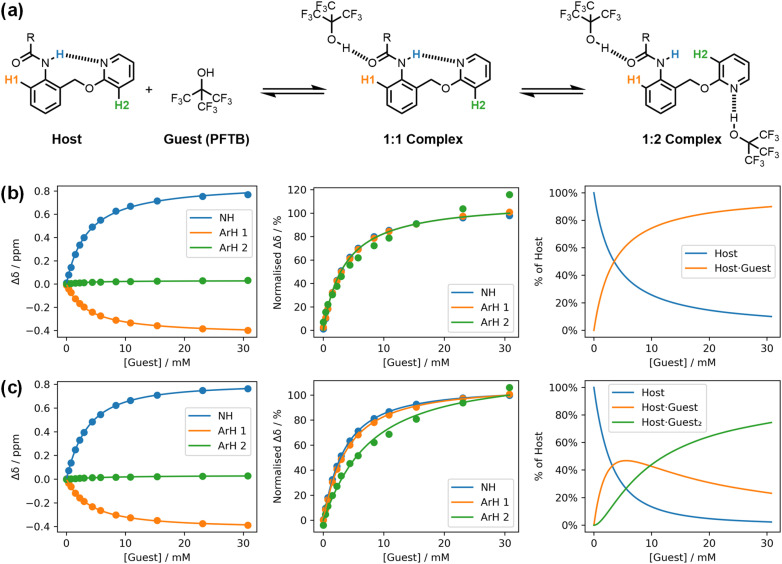
A ^1^H NMR titration (0.208 mM host in *n*-octane at 298 K).^21^ (a) Chemical structures, showing the labelling of the host protons. (b) Best fit to a 1 : 1 binding isotherm (*K* = 293 M^−1^, RMSE = 5.15 × 10^−3^ ppm). (c) Best fit to a 1 : 2 binding isotherm (*K*_1_ = 328 M^−1^, *K*_2_ = 34 800 M^−2^, RMSE = 1.57 × 10^−3^ ppm). Three plots are shown for each model: experimental changes in chemical shift, Δ*δ* (points), and calculated values (lines), normalised changes in chemical shift, and the populations of species that contain host. R = 3-heptyl.

Musketeer provides a number of visualisation tools to facilitate analysis of the quality of fit. In addition to the three types of graphical output illustrated in Fig. 1, there are a number of toggles that change the way in which the data are plotted. Fig. 3 illustrates how normalisation of the changes in chemical shift observed in an NMR titration can be used to compare different models. Three different signals were monitored in a ^1^H-NMR titration using an amide as the host and perfluoro-*tert*-butanol as the guest (Fig. 3a).^21^Fig. 3b shows the fit of the titration data to a 1 : 1 binding isotherm. Visual inspection suggests that this model provides a good description of the data, but replotting the result in a normalised format based on the total change in chemical shift for each signal reveals something different. The green ArH2 data clearly follow a different pattern from the other two signals, which indicates that an additional species is present.

Fig. 3c shows the corresponding plots for a fit to a 1 : 2 binding isotherm. This model clearly accounts for the difference in the behaviour of the three signals and represents a better description of the experimental data. The poor fit of the green data points is masked in the plot of the raw data in Fig. 3b, because the change in chemical shift of this signal is much smaller than the other two signals. However, the green signal is much more sensitive to the second binding event than the other two signals and therefore contains important information for evaluating the different models. Although the association constant obtained from the 1 : 1 model is similar to the value obtained from the 1 : 2 model, the error introduced by using a 1 : 1 binding isotherm leads to an underestimate of the value (293 M^−1^ compared with 328 M^−1^).

## 5 Optimising concentrations as variables

**Fig. 4:**
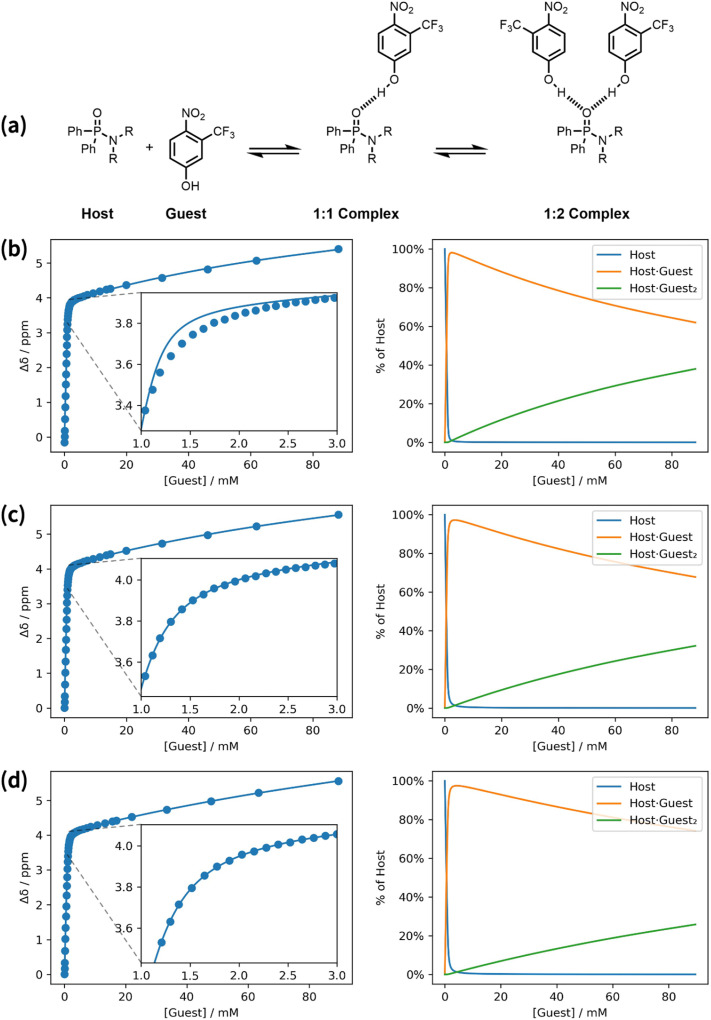
A ^31^P NMR titration carried out close to the tight binding limit (in chlorobenzene at 298 K). (a) Chemical structures. (b) Best fit to a 1 : 2 binding isotherm with the concentrations of host and guest stock solutions fixed at the experimental values of 1.11 mM and 25.0 mM respectively (*K*_1_ = 7.75 × 10^4^ M^−1^, *K*_2_ = 5.48 × 10^5^ M^−2^, RMSE = 6.62 × 10^−2^ ppm). (c) Best fit to a 1 : 2 binding isotherm with the concentration of host stock solution optimised as a variable ([host stock] = 0.95 mM, *K*_1_ = 2.76 × 10^4^ M^−1^, *K*_2_ = 1.51 × 10^5^ M^−2^, RMSE = 5.01 × 10^−3^ ppm). (d) Best fit to a 1 : 2 binding isotherm with the concentration of guest stock solution optimised as a variable ([guest stock] = 29.06 mM, *K*_1_ = 2.45 × 10^4^ M^−1^, *K*_2_ = 9.68 × 10^4^ M^−2^, RMSE = 3.68 × 10^−3^ ppm). Two plots are shown for each model: experimental changes in chemical shift, Δ*δ* (points), and calculated values (lines), and the populations of species that contain host. R = *n*-hexyl.

In general, titrations should be carried out using a host concentration that is less than 10/*K*, to ensure that free host, free guest and the complex are all present in appreciable quantities during the titration. If higher concentrations of host are used, the binding isotherm approaches the limit of two straight lines, rather than a smooth curve, and it becomes impossible to obtain an accurate measurement of the association constant. When a titration is carried out close to this tight binding limit, the quality of the fit is very sensitive to the precise concentrations of the host and guest stock solutions. Fig. 4 shows an example of a ^31^P NMR titration carried out using a higher than usual host concentration (>10/*K*). At the start of the titration, the increase in chemical shift is linear with increasing guest concentration, because every aliquot of guest added binds to the host. When one equivalent of guest has been added, there is an abrupt change in gradient, and the chemical shift increases more gradually as more guest is added, which is indicative a weak second binding event. Fig. 4b shows the result of fitting the titration data using a 1 : 2 binding isotherm. At first sight, the quality of the fit appears reasonable, but closer inspection of the data near the turning point at one equivalent of guest reveals a significant discrepancy. For titrations carried out near the tight binding limit, most of the data points lie on two straight lines, and it is only the curvature of the data points near the turning point that contains information about the association constant. The quality of the fit in this region is therefore critical in these cases.

The fit in Fig. 4b was obtained using fixed concentrations of 1.11 mM for the host stock solution and 25.0 mM for the guest stock, which were determined from the weights of host and guest, and the volume of solvent used in the experiment. The fit in Fig. 4c was obtained by allowing the concentration of the host stock solution to be optimised as a variable. In this case, an excellent fit was obtained at the turning point by using an optimised value of 0.95 mM for the host concentration, which is close to but different from the experimental value. Fig. 4d shows that a similarly good fit can be obtained by allowing the concentration of the guest stock solution to be optimised as a variable instead. The optimised value of 29.1 mM for the guest stock solution is 16% higher than the experimental value, and this percentage increase is the same as the percentage decrease in the optimised value for the host stock solution in the fit in Fig. 4b. In other words, a titration carried out in the tight binding limit accurately identifies the relative values of the host and guest stock solutions, but an error of 16% in either the host solution or the guest solution, or an error of 8% in both, could equally well account for the data.

The association constant obtained from the fit in Fig. 4b (*K*_1_ = 7.75 × 10^4^ M^−1^) is quite different from the values obtained using optimised concentrations (*K*_1_ = 2.76 × 10^4^ M^−1^ or 2.45 × 10^4^ M^−1^ by optimising the host concentration or guest concentration respectively). The variation in association constant obtained by optimising the host or guest concentration (2.6 ± 0.2 × 10^4^ M^−1^) is comparable to the errors obtained from multiple repeats of an NMR titration, whereas the error introduced by not optimising concentration is much larger. The result illustrated in Fig. 4 is a general feature of titrations carried out close to the tight binding limit. An accurate value of the equilibrium constant can only be obtained if the concentration of either the host or guest stock solution is optimised as a variable and a sufficient number of data points is collected in the region of the turning point where there is curvature. Sometimes titrations are intentionally performed close to the tight binding limit in order to verify the stoichiometry of a complex. If the host has *N* guest binding sites, in the tight binding limit the binding isotherm will plateau when the total concentration of guest is *N* times the total concentration of host, allowing *N* to be determined directly from concentration of guest at the turning point. These conditions are widely used for isothermal titration calorimetry (ITC), where a host concentration in the range of 10/*K* to 500/*K* is recommended. In ITC, it is conventional to optimise *N* rather than the concentration of one of the stock solutions, so that discrepancies in the concentrations of the host and guest stock solutions are absorbed in a non-integer value of *N*.^22^ In Musketeer, the same result is achieved by allowing the concentration of the host stock solution to be optimised as a variable.

## 6 Equilibria involving multiple components

**Fig. 5:**
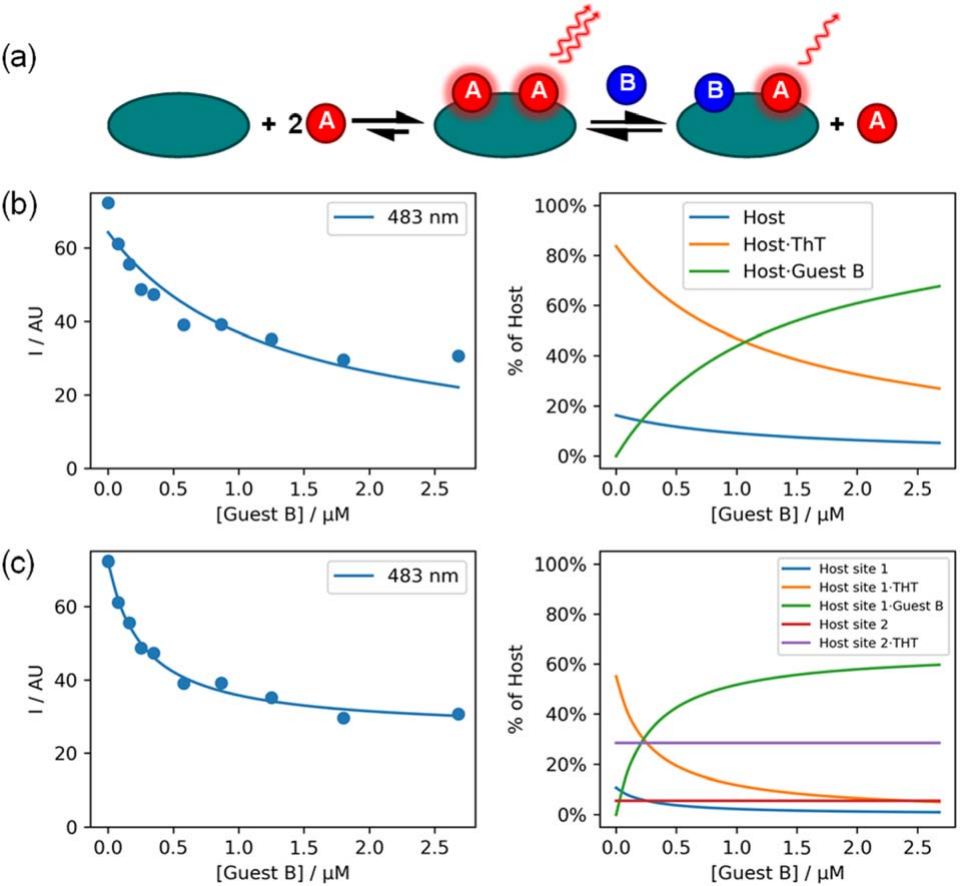
A fluorescence displacement assay showing titration of guest B (E570) into a mixture of host (500 nM Aβ42 fibrils) and guest A (1 μM ThT) in pH 7.4 PBS buffer at 298 K (*λ*_ex_ = 440 nm, *λ*_em_ = 483 nm).^24^ (a) Cartoon representation of the partial displacement model. (b) Best fit to a full displacement model (*K*_B_ = 4.8 × 10^6^ M^−1^, [sites] = 52.4 pM, RMSE = 4.72 AU). (c) Best fit to a partial displacement model (*K*_B_ = 2.3 × 10^7^ M^−1^, [sites] = 81.3 pM, B sites = 66%, RMSE = 1.36 AU). Two plots are shown for each model: experimental fluorescence intensity, *I* (points), and calculated values (lines), and the populations of host species.

The examples above describe simple titration experiments that involve only two components, a host and a guest. However, different types of experiment can involve three or more components. Ligand displacement assays are commonly used in biochemistry to study the interaction of a spectroscopically silent host with a spectroscopically silent guest.^7,23^Fig. 5a shows an example of a fluorescence displacement assay used to investigate the binding of a non-fluorescent ligand (E570) to fibrillar aggregates of the protein Aβ42.^24^ Thioflavin T (ThT) is a ligand that binds to Aβ42 aggregates with a large change in fluorescence emission, so this component was used as guest A, and E570 was titrated as guest B into a mixture of ThT and Aβ42 fibrils. First, the binding constant of the ThT·Aβ42 fibril complex was independently determined in a standard two-component fluorescence titration, and this parameter along with the molar emission of free ThT were used as fixed constants in the analysis of the competition experiment. The three-component titration data were then fit to a model that assumed that E570 completely displaced all of the ThT from the Aβ42 fibrils (Fig. 5b). In this experiment, the concentration of binding sites on the protein aggregates was not known, so this parameter was fitted as a variable, along with the binding constant and the molar emission of the ThT·Aβ42 fibril complex. Visual inspection of the quality of fit in Fig. 5b indicates that this model does not provide a good description of the experimental data, which plateau before all of the ThT is displaced.

Fig. 5c shows the fit obtained using a partial displacement model, which assumes that there are two different binding sites on the fibrils, one of which binds both ThT and E570, and one that binds only ThT. Although this model requires the relative proportions of the two binding sites as an additional variable, visual inspection of the quality of fit confirms that this model gives a significantly better description of the data.

## 7 Reducing the number of variables

**Fig. 6:**
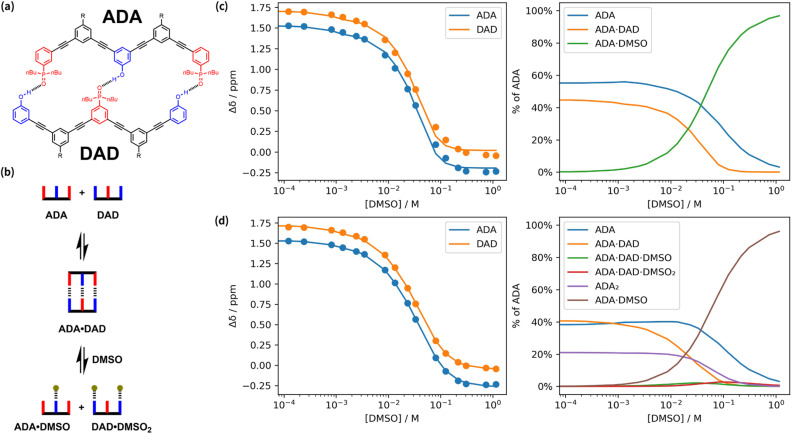
A ^31^P NMR denaturation experiment showing addition of DMSO to a mixture of two oligomers, ADA and DAD, in chloroform at 298 K.^25^ (a) Structure of the H-bonded duplex formed by ADA and DAD. (b) Cartoon representation of the species present in a three-state denaturation model. (c) Best fit to the three state denaturation model in (b) (*K*_ADA·DAD_ = 1,130 M^−1^, RMSE = 4.10 × 10^−2^ ppm). (d) Best fit to the more complicated model illustrated in Fig. 7 and 8 (*K*_ADA·DAD_ = 2150 M^−1^, RMSE = 1.10 × 10^−2^ ppm). Two plots are shown for each model: experimental difference in chemical shift compared with a reference phosphine oxide at the same concentration of DMSO, Δ*δ* (points), and calculated values (lines), and the populations of species that contain ADA. R = CO_2_^*i*^Bu.

Fitting titration data for complicated equilibria that involve multiple species generally requires optimisation of a large number of different variables. In the competition experiment above, it was possible to eliminate some of the potential variables by independently measuring pairwise interactions between two of the components of the three-component system. An alternative strategy implemented in Musketeer is to reduce the number of variables by setting up mathematical relationships between parameters. Fig. 6a shows the structures of two oligomers, ADA and DAD, that form a H-bonded duplex.^25^ The ADA·DAD complex was studied using a ^31^P NMR denaturation experiment, in which DMSO was titrated into a mixture of the two oligomers. The model in Fig. 6b, which assumes that the oligomers could be free, bound in the duplex or bound to DMSO, was initially used to fit the data. The association constant for the phenol·DMSO complex (*K*_DMSO_) was measured independently by a ^1^H NMR titration experiment, and this value was used to fix the equilibrium constants for formation of the denatured species, ADA·DMSO and DAD·DMSO_2_, as *K*_DMSO_ and *K*^2^_DMSO_ respectively. Fig. 6c shows the best fit to the model in Fig. 6b, which was obtained by optimising the free and bound phosphine oxide chemical shifts and the equilibrium constant for duplex formation. The three state model does not describe the denaturation data well at high DMSO concentrations, which suggests that additional species, such as partially denatured intermediates, should be included.

**Fig. 7:**
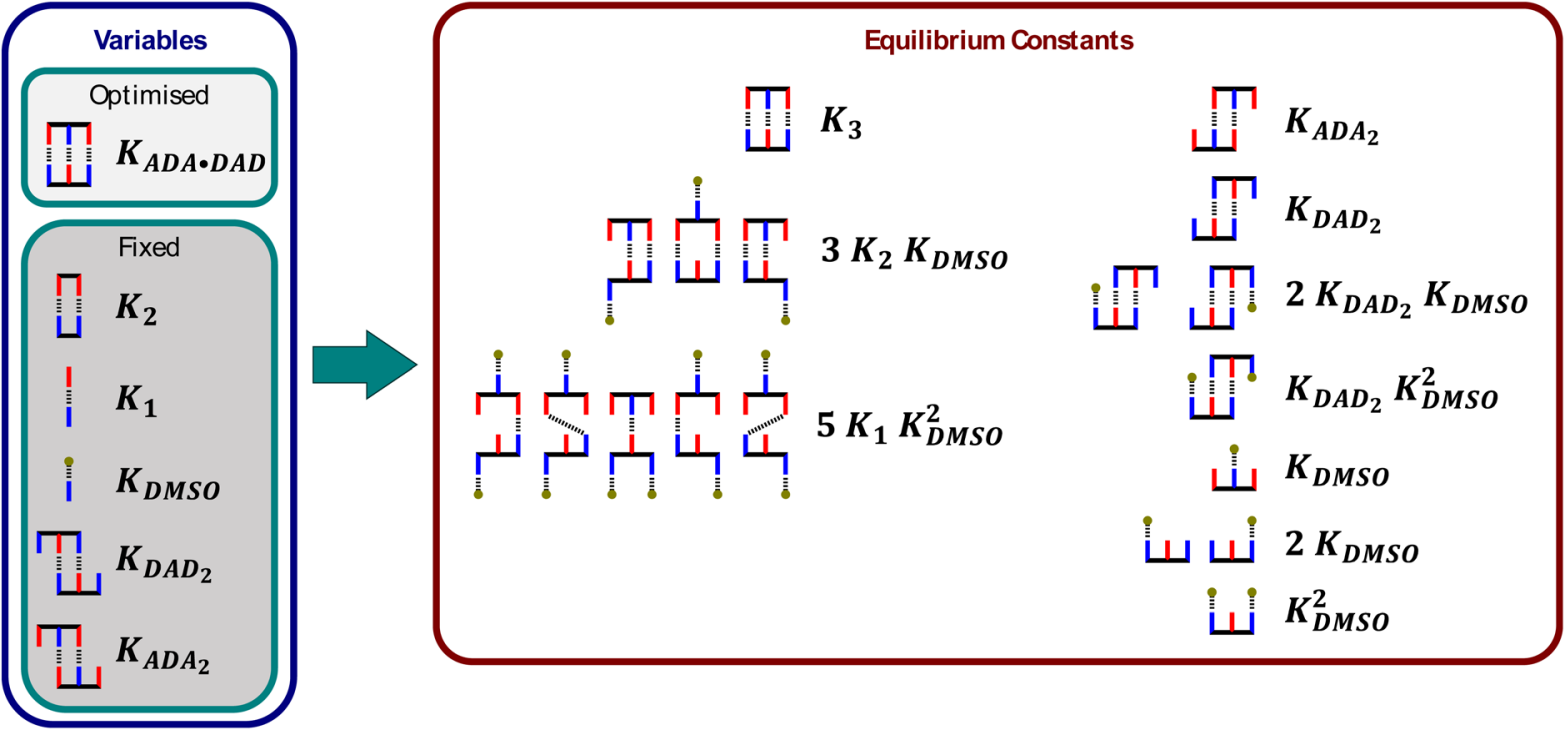
Cartoon representation of all possible species present in the DMSO denaturation of ADA·DAD (the grey balls represent DMSO). The equilibrium constants of nine of the ten species are expressed as a function of five known equilibrium constants. Degenerate binding modes are shown for each stoichiometry, and the relevant statistical factors are included in the equilibrium constants.

Fig. 7 shows that there are ten different stoichiometric species that could be present in this system (degenerate binding modes are shown for each stoichiometry), and a different equilibrium constant and chemical shift is required to describe each one. Although some of these parameters can be independently determined, there are too many unknowns for any fit of the denaturation data to be reliable. This problem can be solved be making some assumptions about relationships between the parameters to dramatically reduce the number of variables without reducing the complexity of the model. Fig. 6 shows that the equilibrium constants required to describe the ten different stoichiometric species can be expressed in terms of six different equilibrium constants, five of which can be determined experimentally, leaving just one variable, the ADA·DAD association constant *K*_ADA·DAD_. The association constants for duplexes that make one and two H-bonds (*K*_1_ and *K*_2_) were measured by ^31^P NMR titration experiments using shorter oligomers, and the association constants for ADA_2_ and DAD_2_ were measured by ^31^P NMR dilution experiments.

**Fig. 8:**
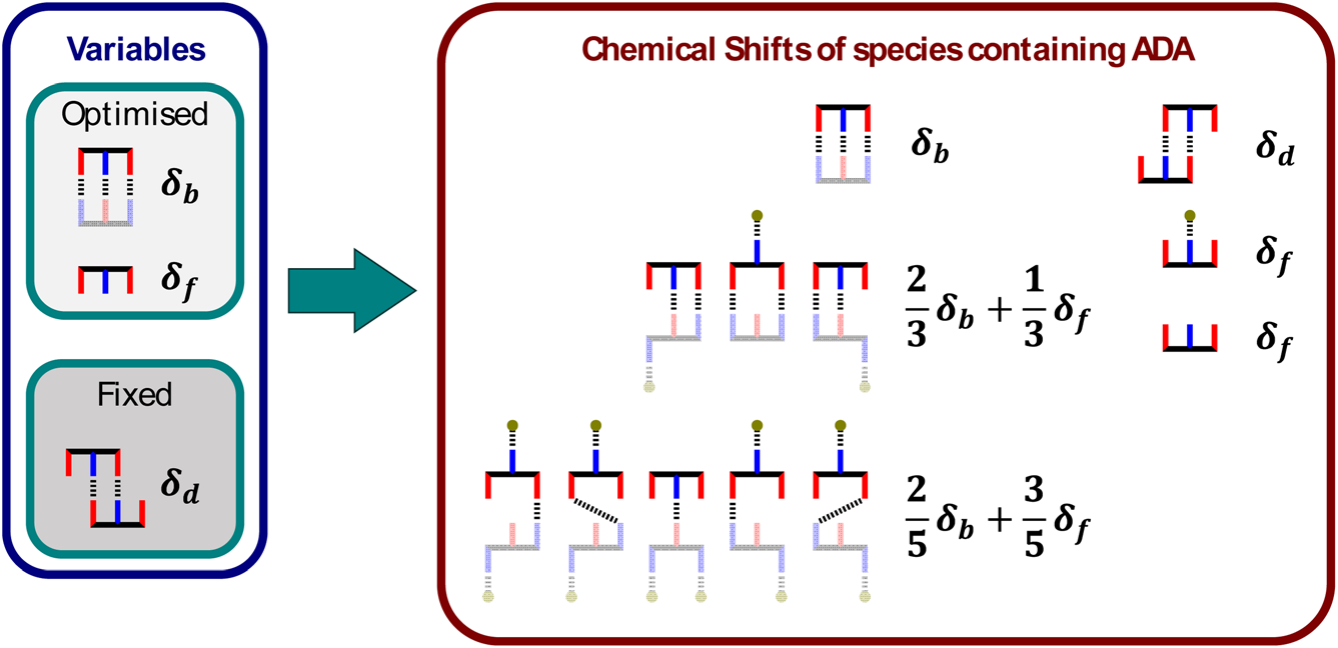
Cartoon representation of all ADA species present in the DMSO denaturation of ADA·DAD (the DAD components that do not contribute to the chemical shift of the ADA signal are greyed out). The chemical shifts of five of the six species are expressed as a function of two variables, and the chemical shift of ADA_2_ was independently measured (*d*_d_). Degenerate binding modes are shown for each stoichiometry.

A similar approach was used to reduce the number of variables required to describe the chemical shifts of the ten different stoichiometric species. The chemical shifts of ADA_2_ and DAD_2_ were measured directly by ^31^P NMR dilution experiments, and the chemical shifts of all other species were treated as the population-weighted average of signals due to free and bound phosphine oxide groups. Fig. 8 shows how the chemical shifts of all species involving ADA are described using just two variables, *δ*_f_ and *δ*_b_. Similar relationships can be written for the species involving DAD using two analogous variables for the chemical shifts. Fig. 6d shows the fit to the DMSO denaturation data that was obtained using this model. The quality of the fit in Fig. 6d is significantly better than the fit in Fig. 6c, even though the same number of variables were optimised: the free and bound phosphine oxide chemical shifts, and the association constant for duplex formation.

## 8 The algorithm

### 8.1. Linear and nonlinear variables

Fitting titration data can involve finding the optimum values for a large number of different variables. For UV/vis absorption titration data recorded at 300 wavelengths, fitting to a 1 : 1 binding isotherm with a spectroscopically silent guest involves 601 variables: the equilibrium constant, and the free and bound molar absorption coefficients at each wavelength. If these variables are optimised simultaneously, fitting will take a long time, and there is a high risk that the result will be a local minimum rather than the optimal values for all variables. To increase the speed of fitting and avoid local minima, we first separate the linear and nonlinear variables. Unknown total concentrations of the components and equilibrium constants are nonlinear variables. However, given the values of those variables, the concentrations of all species present at each addition can be calculated (see speciation algorithm below), and from there the concentrations of all spectroscopically active states are obtained by a simple linear transformation. The observed signal is then given by

3 ***Y*** = ***AX***

where ***Y*** is the matrix of the observed spectra with dimensions of number of additions and number of wavelengths, ***A*** is the matrix of the concentrations of all spectroscopically active states with dimensions of number of additions and number of states, and ***X*** is the matrix of variables to be optimised, namely the molar absorption coefficients of all spectroscopically active states with dimensions of number of states and number of wavelengths.

Given ***Y*** and ***A***, the exact solution for the linear variables ***X*** can quickly be found using linear regression. By separating the variables this way, the fitting can be reformulated as a bilevel optimisation problem. The objective function to be optimised depends only on the nonlinear variables. For each input value, the objective function calculates ***A***, solves for ***X***, and returns the RMSE of the solution. A nonlinear optimisation algorithm can then be used to find the values for the nonlinear variables that return the smallest RMSE. In Musketeer, the Nelder–Mead method^26^ is used for the nonlinear optimisation, as implemented in the SciPy package.^27^

### 8.2. Speciation

The most computationally expensive step of the optimisation process is calculation of the concentrations of all species at each addition given the total concentrations and equilibrium constants, *i.e.* the speciation. For some common binding isotherms, such as 1 : 1 complexes or polymers of a single component, closed-form solutions can easily be found. However, for more complicated models with multiple competing equilibria, an exact solution usually requires finding the roots of a high order polynomial, and deriving the precise form of this polynomial may not be computationally feasible. Instead, it is usually quicker to solve the speciation for a complicated isotherm numerically to the desired precision. The speciation algorithm used by Musketeer is described below, and the matrix notation is explained in Table 1 using formation of a 1 : 2 complex as an example.

**Table 1:** Symbols used in the speciation algorithm

Matrix	Meaning	Example for a 1 : 2 isotherm
***s***	Concentrations of free components	([H] : [G])
***c***	Concentrations of complexes	([HG] : [HG_2_])
***t***	Total concentrations of components	([H]_0_ : [G]_0_)
***β***	Global equilibrium constants for formation of complexes	(*K*_HG_ : *K*_HG_2__)
***M***	Stoichiometries of complexes (rows are components, columns are complexes)	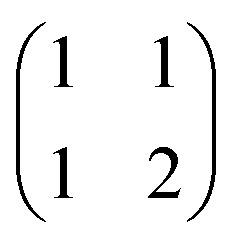

The speciation algorithm must determine ***s*** and ***c***, given ***t***, ***β***, and ***M***. Mass balance means that the total concentration of each component is equal to the concentration of the free component plus the concentration of each complex multiplied by the stoichiometric coefficient of the component in that complex. This gives the following constraint:

4 ***t*** = ***s*** + ***Mc***

The concentration of each complex *c*_*j*_ is given by the corresponding global equilibrium constant multiplied by the product of the concentration of each component raised to the power of the stoichiometric coefficient:5
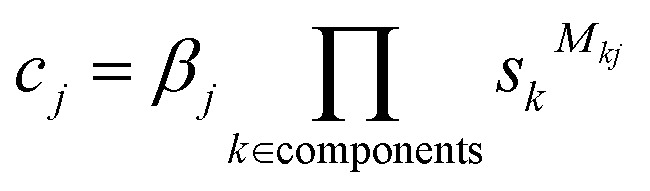


Substituting eqn (5) into (4) gives the following set of constraints ***f*** = 0:6
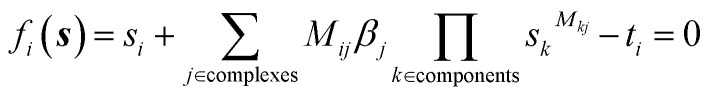


Solving for the value of ***s*** that satisfies all constraints in ***f*** = 0 will give the concentrations of all free components at equilibrium, and the concentrations of all complexes can then be calculated using eqn (5). Rather than trying to solve all constraints simultaneously, the process can be simplified by first noting that7
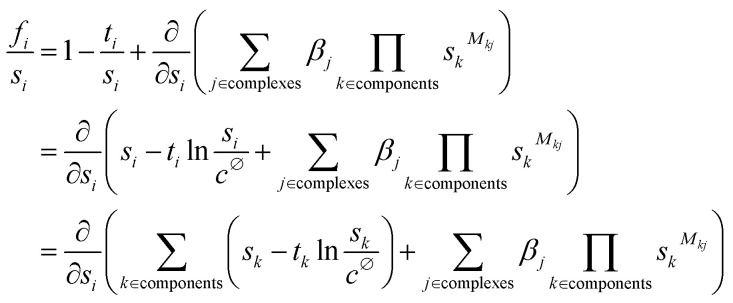
where *c*^∅^ = 1 M is introduced to preserve units inside the logarithm.

Eqn (7) shows that the set of constraints ***f*** can be expressed as the partial derivatives of a single multivariate function, *F*(***s***), which is defined in eqn (8) (the corresponding function for polymeric species is described in the ESI†).8
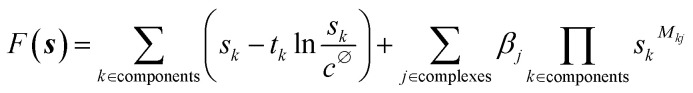


Therefore, satisfying all constraints ***f*** = 0 is equivalent to solving for ∇*F*(***s***) = 0, *i.e.* finding the minimum of *F*(***s***). Since there is only one set of concentrations at which a system will be at equilibrium, *F*(***s***) has no local minima, and so a numerical optimisation method can be used to find the minimum. By calculating appropriate boundary conditions to ensure numerical stability, this function can be guaranteed to converge to any desired precision for any system (see ESI†). In Musketeer, the fastest results were obtained by using the L-BFGS-B algorithm^28^ as implemented in the SciPy package.^27^

## 9 Conclusions

Musketeer is a versatile software tool that can be used for the analysis of data from a range of different types of titration experiment. There are no constraints on the spectroscopic technique or the complexity of the binding isotherm. The quality of the fit obtained using different models can be rapidly evaluated without the need to manually derive the equations for complicated binding isotherms. The resulting analysis can then be shared as a single.fit file, allowing others to easily verify the fit to any model, or adapt the model for their own experiments. The software can be easily installed on macOS and Windows computers and has been made freely available on both GitHub and PyPI. A step-by-step guide to using Musketeer is also provided as ESI.†

## Author contributions

The manuscript was written through contributions of all authors.

## Conflicts of interest

There are no conflicts to declare.

## Supplementary Material

SC-015-D4SC03354J-s001

SC-015-D4SC03354J-s002

## Data Availability

All supporting data is provided in the ESI.†
