# Supplementary material for: Musketeer: a software tool for the analysis of titration data
Source: Chem Sci. 2024 Aug 29;15(37):15299–310. doi: 10.1039/d4sc03354j (PMC11376196; doi:10.1039/d4sc03354j)
Supplement: SC-015-D4SC03354J-s002 [file SC-015-D4SC03354J-s002.pdf]

Supplementary Information (SI) for Chemical Science.
This journal is © The Royal Society of Chemistry 2024

Musketeer: a Software Tool for the Analysis of Titration Data

Daniil O. Soloviev and Christopher A. Hunter*

Yusuf Hamied Department of Chemistry, University of Cambridge, Lensfield Road, Cambridge CB2 1EW, UK

Supplementary Information

Musketeer Worked Example

The duplex denaturation experiment shown in Figures 6–8 of the main text will be used as a worked example of how to implement complicated models in Musketeer. A CSV file containing the spectroscopic data and the concentrations of the three components after each addition is provided as Supporting Information.

When a new fit is started in Musketeer, the user interface opens with two panels. The panel on the left is used to set up the fit, i.e. enter the experimental data, specify equilibria to be used in the model, and define the relationship between the species present and the spectra. The panel on the right displays the results of fitting.

Experiment: Enter spectroscopic data

In the ”Experiment” section, the “Enter/edit spectroscopic data” button brings up a window with a spreadsheet interface (Figure S1). The two columns in the CSV file that contain the chemical shift data can be copied and pasted into the popup window, as shown in Figure S1. The “Signal titles” checkbox should be selected to indicate that the first row of the spreadsheet contains labels for the signals, ADA and DAD. The “Measured quantity” and “Unit” should be specified as “Δδ” and “ppm” respectively to ensure that the output graphs are correctly labelled. Finally, the “OK” button saves the changes and closes the popup window.

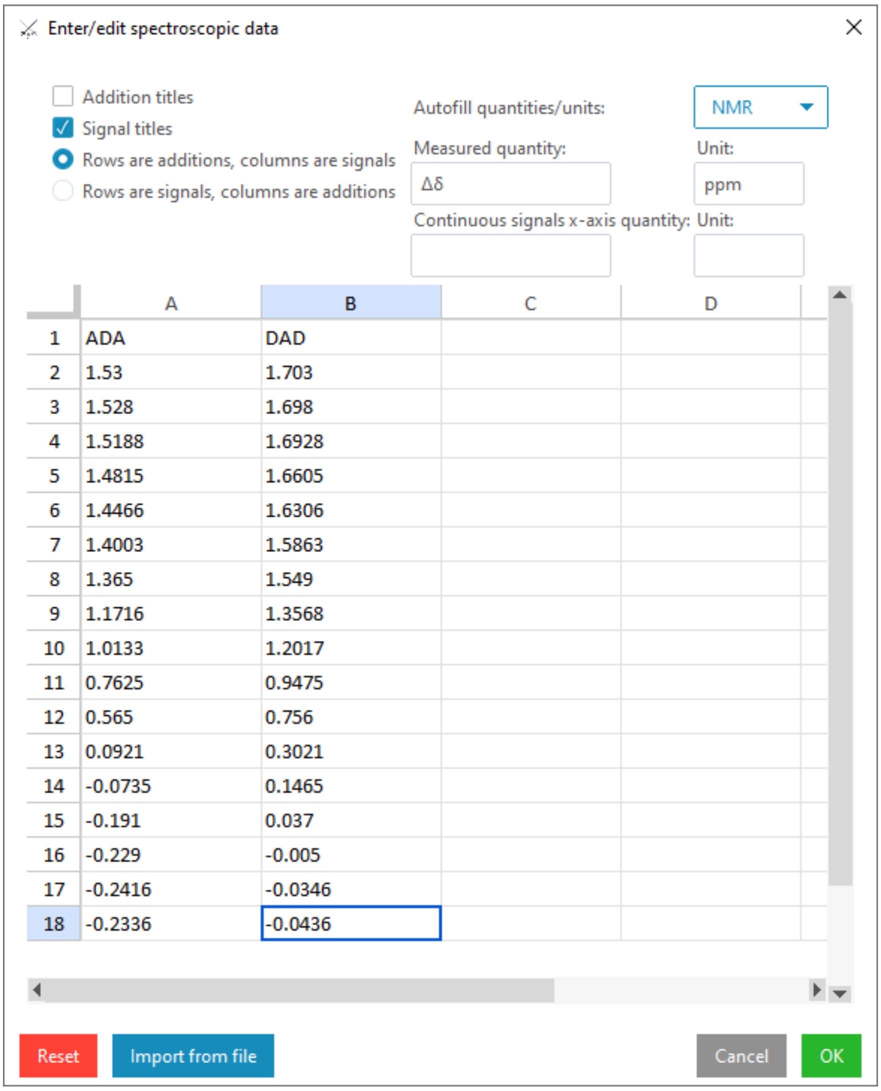

Figure S1. Popup window for entering spectroscopic data in Musketeer.

Experiment: Enter concentrations

The next dropdown menu is used to enter the concentrations of all components after each addition. The "Concentrations" option brings up a new popup window (Figure S2). By default, two components are listed in this window, so the "New column" button must be used to add an extra column, because the denaturation experiment involves three different components. A label should be entered for each component, in this case, ADA, DAD and DMSO. The concentrations from the CSV file can then be copied and pasted into the popup window to populate the table, as shown in Figure S2. The units of concentration must be specified from the dropdown list (M), and the "OK" button is used to save the data.

It is also possible to optimise any number of concentrations as variables. To do this, "?" can be entered in a cell, or "~value" to provide an initial guess for the optimisation. This can also be done for the concentrations of stock solutions, when entering addition volumes rather than concentrations directly. In both cases, a checkbox is provided if multiple concentrations need to be optimised as a single variable.

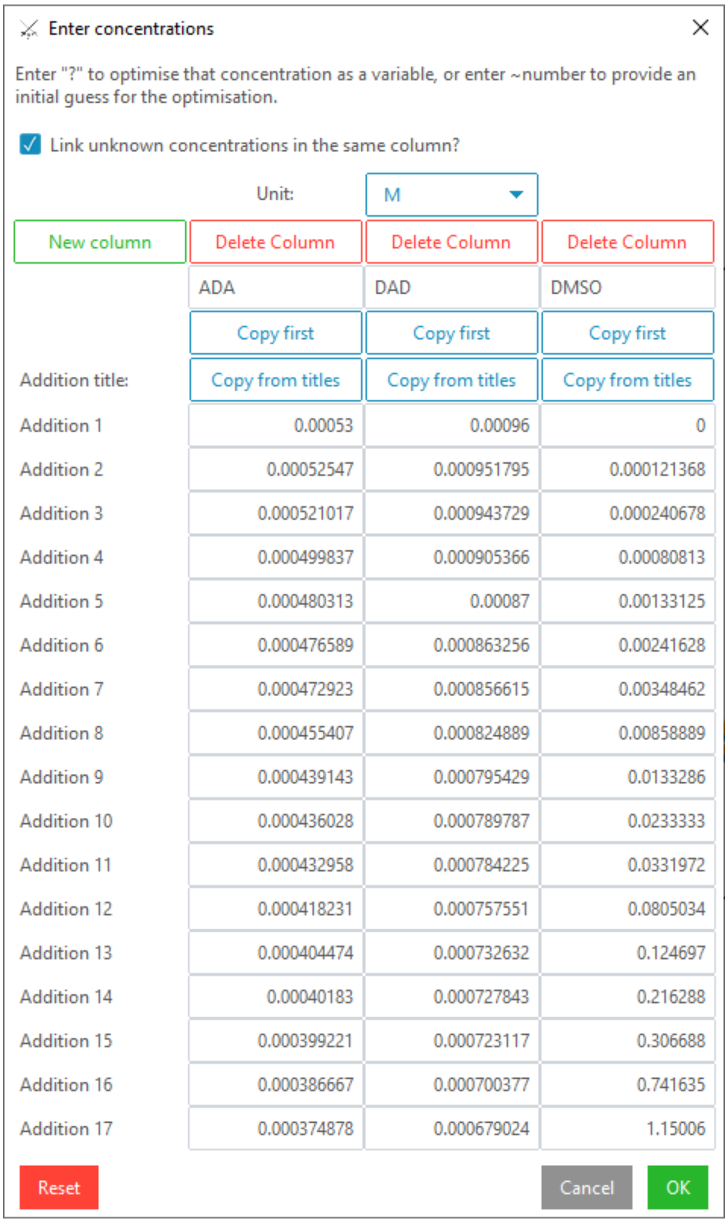

Figure S2. Popup window for entering concentrations in Musketeer.

Experiment: Specify fast/slow exchange

The last dropdown in the "Experiment" section is used to specify whether the spectroscopic signals are proportional to concentration (slow exchange) or mole fraction (fast exchange). For the NMR denaturation experiment, "Mole fraction (fast exchange)" is used.

Equilibria: Select a binding isotherm

The "Equilibria" section is then used to define the model to be used to fit the data. The denaturation experiment involves multiple competing equilibria, so the "Custom" option must be selected from the dropdown menu under "Select a binding isotherm". The popup window is used to specify the stoichiometries of all species, which appear as rows. By default, there is one row for each free component and one row for a 1:1 complex between the first two components. To specify all ten complexes shown in Figure 7 of the main text, nine additional rows must be added. The stoichiometry of each species is entered as the number of molecules of each component, as shown in Figure S3. A label is automatically generated for each complex, which can be used to verify that the stoichiometries have been entered correctly. The speciation table is saved using the "OK" button.

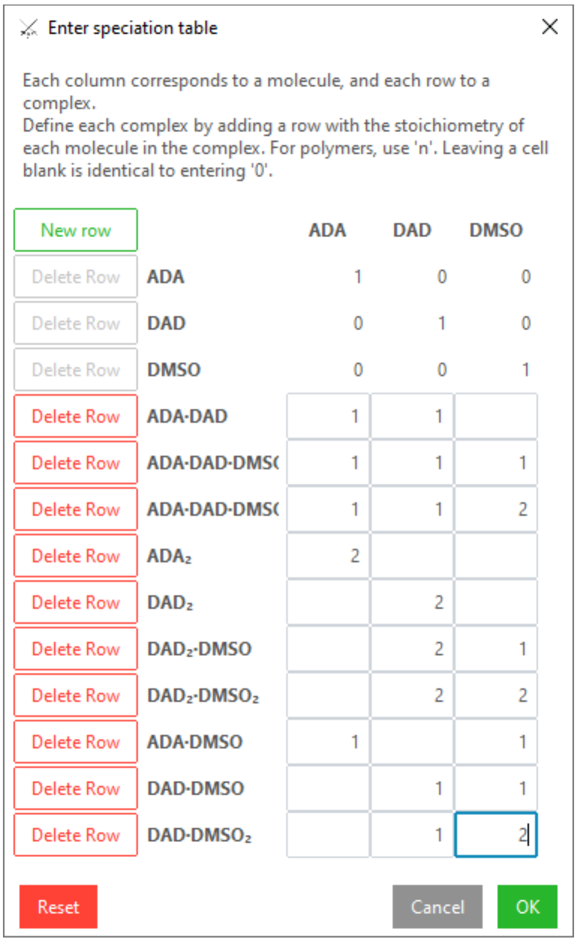

Figure S3. Popup window for entering stoichiometries in Musketeer.

Equilibria: Fix any K values

The next dropdown menu, "Fix any K values", is used to reduce the number of variables. Selecting "Custom" brings up a window with a new table, where the rows specify a set of parameters, and the columns correspond to the complexes defined in the speciation table (Figure S4). This window is used to enter the relationships between the equilibrium constants defined in Figure 7 of the main text. The first row is used to enter statistical factors that describe the degeneracies of the complexes. The global equilibrium constant for each complex is defined as the product of the statistical factor and the parameter in each row raised to the power of the entry in the relevant column of the table. By default, the table appears as one row and one column for each complex, with ones along the diagonal, and zeros everywhere else, so that the global equilibrium constant for each complex would be equal to one of the parameters defined by the rows. To implement the model shown in Figure 7 of the main text, the global equilibrium constants for the ten complexes should be defined in terms of six different parameters. Therefore, four of the rows should be deleted from the table and the remaining six rows defined as K_{DMSO}, K_{ADA2}, K_{DAD2}, K_1, K_2 and $K_{ADA•DAD}$. The cells in the table are then used to specify the mathematical relationship between the global equilibrium constant for each of the complex and the six parameters (Figure S4). The equations defining the global equilibrium constants are automatically displayed below the table and can be used to verify that the relationships have been entered correctly. The last column of the table allows any known values of the parameters to be fixed by entering the relevant value, or optimised in the fitting process by entering "?".

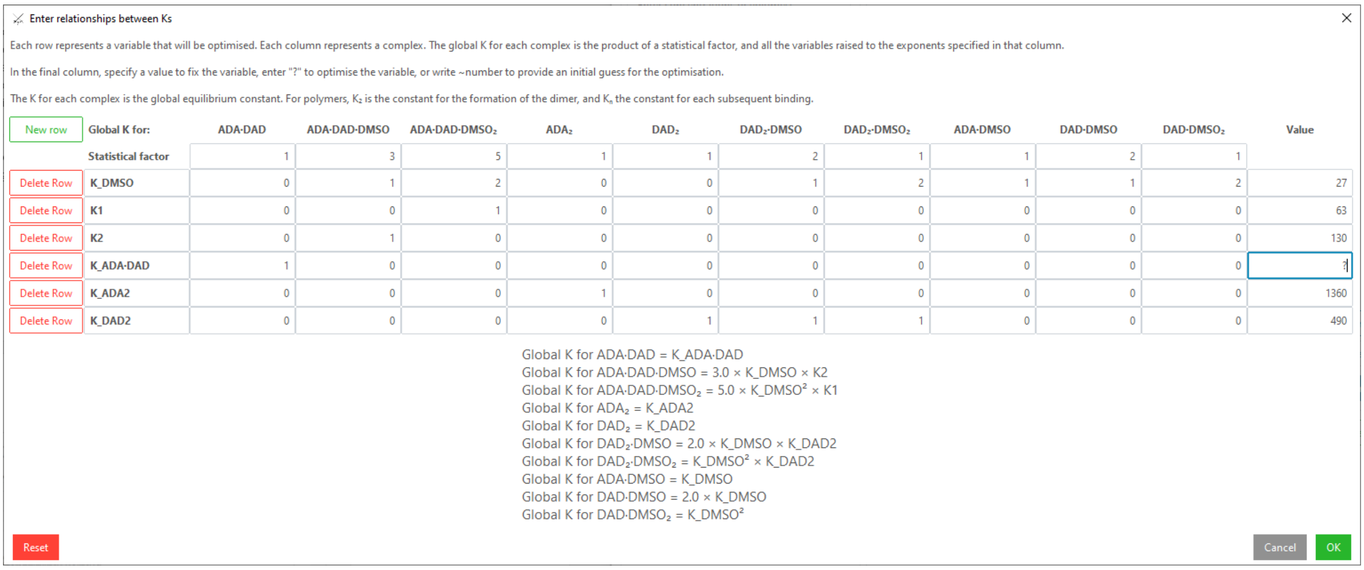

Figure S4. Popup window for describing relationships between equilibrium constants in Musketeer.

Spectra: Which species contribute to the spectra

The "Spectra" section is used to describe how the various species contribute to the spectra. The first dropdown menu is used to specify "Which species contribute to the spectra". In this case, there are two different NMR signals due to two different components, ADA and DAD, so the "Custom, different per signal" must be used to ensure that only the species containing the relevant component are included in the calculation of mole fractions for the fast exchange signals. Each signal is assigned to the corresponding component as shown in Figure S5.

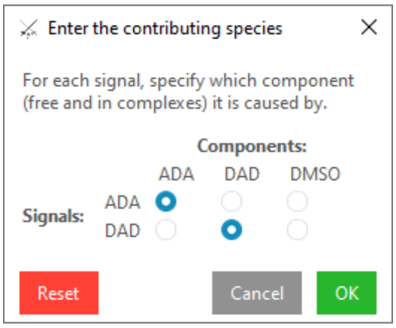

Figure S5. Popup window for specifying which components contribute to which signals in Musketeer.

Spectra: Specify relationship between fitted spectra

The next dropdown menu is used to implement the chemical shift relationships shown in Figure 8 of the main text. Selecting “Custom” from the “Specify relationship between fitted spectra” dropdown generates a popup window with a table for each spectroscopically active component. There is a column for each species that contains the relevant component, and the rows define the different states that contribute to the signal. In this model, the ADA phosphine oxide groups can take three different states (free, bound or homodimer), so three rows are needed in the table. The cells in the table specify how many times each state appears in each species. For example, free ADA contains two phosphine oxides in the free state, whereas ADA in the ADA•DAD•DMSO complex contains 4/3 phosphine oxides in the bound state and 2/3 of a phosphine oxide in the free state. Full details of how enter the chemical shift relationships for ADA and for DAD are shown in Figure S6.

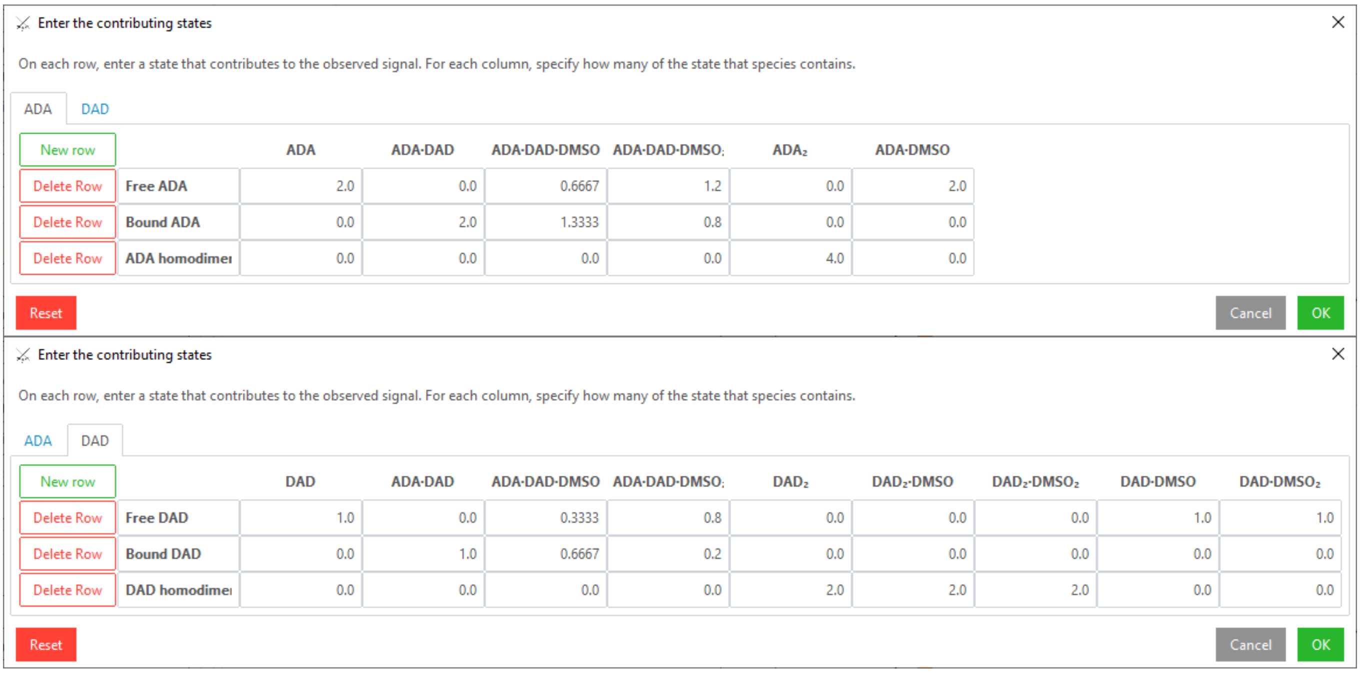

Figure S6. Popup window for describing how different states contribute to the observed spectroscopic signals in Musketeer.

Spectra: Specify any known spectra

Known values for any spectra can be fixed using the dropdown menu "Specify any known spectra". In the denaturation experiment, the chemical shift changes for the homodimers are known from dilution experiments, so these values can be set to 2.0 and 4.3 ppm, as shown in Figure S7. The remaining cells are left as "?" to be optimised as variables.

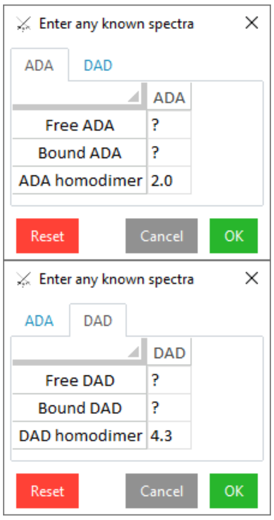

Figure S7. Popup window for specifying known spectra in Musketeer.

Fit data

Once the model has been entered, pressing the "Fit" button finds optimal values for all of the variables, and creates three new tabs on the screen displaying the results: the experimental data points and the calculated lines of the best fit, the calculated populations of all the species, and the values of all optimised variables. If the user wants to explore a slightly different model, the "Copy fit" button can be used to create a new tab, which contains a duplicate of the model that can be modified and fitted independently. Once a satisfying fit is obtained, the File menu at the top of the screen is used to save all tabs as a .fit file, which can be used to review or share the fit. The .fit file for this denaturation experiment is included in the Supporting Information.

The Musketeer Algorithm

Linear and nonlinear variables

Fitting titration data can involve finding the optimum values for a large number of different variables. For UV/Vis absorption titration data recorded at 300 wavelengths, fitting to a 1:1 binding isotherm with a spectroscopically silent guest involves 601 variables: the equilibrium constant, and the free and bound extinction coefficients at each wavelength. If these variables are optimised simultaneously, fitting will take a long time, and there is a high risk that the result will be a local minimum rather than the optimal values for all variables. To increase the speed of fitting and avoid local minima, we first separate the linear and nonlinear variables. Unknown total concentrations of the components and equilibrium constants are nonlinear variables. However, given the values of those variables, the concentrations of all species present at each addition can be calculated (see speciation algorithm below), and from there the concentrations of all spectroscopically active states are obtained by a simple linear transformation. The observed signal is then given by

$$\boldsymbol{Y} = \boldsymbol{A}\boldsymbol{X} \tag{1}$$

where $\boldsymbol{Y}$ is the matrix of the observed spectra with dimensions of number of additions and number of wavelengths, $\boldsymbol{A}$ is the matrix of the concentrations of all spectroscopically active states with dimensions of number of additions and number of states, and $\boldsymbol{X}$ is the matrix of variables to be optimised, namely the molar extinction coefficients of all spectroscopically active states with dimensions of number of states and number of wavelengths.

Given $\boldsymbol{Y}$ and $\boldsymbol{A}$, the exact solution for the linear variables $\boldsymbol{X}$ can quickly be found using linear regression. By separating the variables this way, the fitting can be reformulated as a bilevel optimisation problem. The objective function to be optimised depends only on the nonlinear variables. For each input value, the objective function calculates $\boldsymbol{A}$, solves for $\boldsymbol{X}$, and returns the RMSE of the solution. A nonlinear optimisation algorithm can then be used to find the values for the nonlinear variables that return the smallest RMSE. In Musketeer, the Nelder-Mead method[1] is used for the nonlinear optimisation, as implemented in the SciPy package.[2]

Speciation algorithm

The most computationally expensive step of the optimisation process is calculation of the concentrations of all species at each addition given the total concentrations and equilibrium constants, i.e. the speciation. For some common binding isotherms, such as 1:1 complexes or polymers of a single component, closed-form solutions can easily be found. However, for more complicated models with multiple competing equilibria, an exact solution usually requires finding the roots of a high order polynomial, and deriving the precise form of this polynomial may not be computationally feasible. Instead, it is usually quicker to solve the speciation for a complicated isotherm numerically to the desired precision. The speciation algorithm used by Musketeer is described below, and the matrix notation is explained in Table 1 using formation of a 1:2 complex as an example.

Matrix	Meaning	Example for a 1:2 isotherm
$\boldsymbol{s}$	Concentrations of free components	$([H] \quad [G])$
$\boldsymbol{c}$	Concentrations of complexes	$([HG] \quad [HG_2])$
$\boldsymbol{t}$	Total concentrations of components	$([H]_0 \quad [G]_0)$
$\boldsymbol{\beta}$	Global equilibrium constants for formation of complexes	$(K_{HG} \quad K_{HG_2})$
$\boldsymbol{M}$	Stoichiometries of complexes<br>(rows are components, columns are complexes)	$\begin{pmatrix} 1 & 1 \\ 1 & 2 \end{pmatrix}$

Table 1: Symbols used in the speciation algorithm.

The speciation algorithm must determine $\boldsymbol{s}$ and $\boldsymbol{c}$, given $\boldsymbol{t}$, $\boldsymbol{\beta}$, and $\boldsymbol{M}$. Mass balance means that the total concentration of each component is equal to the concentration of the free component plus the concentration of each complex multiplied by the stoichiometric coefficient of the component in that complex. This gives the following constraint:

$$\boldsymbol{t} = \boldsymbol{s} + \boldsymbol{Mc} \tag{2}$$

The concentration of each complex c_j is given by the corresponding global equilibrium constant multiplied by the product of the concentration of each component raised to the power of the stoichiometric coefficient:

$$c_j = \beta_j \prod_{k \in \text{components}} s_k^{M_{kj}} \tag{3}$$

Substituting Equation (3) into (2) gives the following set of constraints $\boldsymbol{f} = \boldsymbol{0}$:

$$f_i(\boldsymbol{s}) = s_i + \sum_{j \in \text{complexes}} M_{ij}\beta_j \prod_{k \in \text{components}} s_k^{M_{kj}} - t_i = 0 \tag{4}$$

Solving for the value of $\boldsymbol{s}$ that satisfies all constraints in $\boldsymbol{f} = \boldsymbol{0}$ will give the concentrations of all free components at equilibrium, and the concentrations of all complexes can then be calculated using Equation (3). Rather than trying to solve all constraints simultaneously, the process can be simplified by first noting that

$$\begin{aligned} \frac{f_i}{s_i} &= 1 - \frac{t_i}{s_i} + \frac{\partial}{\partial s_i}\left(\sum_{j \in \text{complexes}} \beta_j \prod_{k \in \text{components}} s_k^{M_{kj}} \right) \\ &= \frac{\partial}{\partial s_i}\left(s_i - t_i \ln\frac{s_i}{c^{\ominus}} + \sum_{j \in \text{complexes}} \beta_j \prod_{k \in \text{components}} s_k^{M_{kj}} \right) \\ &= \frac{\partial}{\partial s_i}\left(\sum_{k \in \text{components}} \left(s_k - t_k \ln\frac{s_k}{c^{\ominus}} \right) + \sum_{j \in \text{complexes}} \beta_j \prod_{k \in \text{components}} s_k^{M_{kj}} \right) \end{aligned} \tag{5}$$

where $c^{\ominus} = 1$ M is introduced to preserve units inside the logarithm.

Equation (5) shows that the set of constraints $\boldsymbol{f}$ can be expressed as the partial derivatives of a single multivariate function, $F(\boldsymbol{s})$, which is defined as

$$F(\boldsymbol{s}) = \sum_{k\in\text{components}} \left(s_k - t_k \ln\frac{s_k}{c^{\ominus}}\right) + \sum_{j\in\text{complexes}} \beta_j \prod_{k\in\text{components}} s_k^{M_{kj}} \tag{6}$$

Therefore, satisfying all constraints $\boldsymbol{f} = \boldsymbol{0}$ is equivalent to solving for $\boldsymbol{\nabla}F(\boldsymbol{s}) = \boldsymbol{0}$, i.e. finding the minimum of $F(\boldsymbol{s})$. Since there is only one set of concentrations at which a system will be at equilibrium, $F(\boldsymbol{s})$ has no local minima, and so a numerical optimisation method can be used to find the minimum. In Musketeer, the fastest results were obtained by using the L-BFGS-B algorithm[3] as implemented in the SciPy package.[2]

In order guarantee convergence to any desired precision and make the optimisation independent of the order of magnitude of the concentrations, we can introduce a change of variables. Rather than optimising $F(\boldsymbol{s})$ directly with respect to $\boldsymbol{s}$, we define a new vector $\boldsymbol{x}$ as

$$\boldsymbol{x} = \boldsymbol{t} \odot \ln\frac{\boldsymbol{s}}{c^{\ominus}} \tag{7}$$

where $\odot$ is the Hadamard product.

This change of variables allows $F(\boldsymbol{s})$ to be transformed into a new function $G(\boldsymbol{x})$, defined as

$$G(\boldsymbol{x}) = F(c^{\ominus} \exp(\boldsymbol{x} \oslash \boldsymbol{t})) = F(\boldsymbol{s}) \tag{8}$$

where $\oslash$ is Hadamard division.

By differentiating Equation (8) with respect to $\boldsymbol{x}$ and substituting for $F(\boldsymbol{s})$ from Equation (5) and $\boldsymbol{x}$ from Equation (7), we can see that the gradient of $G(\boldsymbol{x})$ is the relative error in the total concentration of each component:

$$\boldsymbol{\nabla}G(\boldsymbol{x})_i = \frac{\partial}{\partial x_i}G(\boldsymbol{x}) = \frac{\dfrac{\partial F(\boldsymbol{s})}{\partial s_i}}{\dfrac{\partial x_i}{\partial s_i}} = \frac{\dfrac{f_i}{s_i}}{\dfrac{t_i}{s_i}} = \frac{f_i}{t_i} = \frac{s_i + \sum_{j\in\text{complexes}} M_{ij}\, c_j - t_i}{t_i} \tag{9}$$

Therefore, the criteria for convergence of the numerical minimisation can be set to each component of the gradient being equal to or less than the desired relative precision in the mass balance. To avoid division by zero, if the total concentration t_i of any component is zero, then s_i must also be zero, and that component is excluded from the minimisation.

To ensure numerical stability, boundary conditions must be provided for the optimisation. For small values of x_i, which correspond to s_i approaching zero, $G(\boldsymbol{x})$ can exceed the range of representable floating-point numbers and cause the line search step of the minimisation to fail. Therefore, boundary conditions must be provided to restrict the range of values sampled in the optimisation process. For component i, t_i is the largest physically meaningful value for s_i, so this value is used as the upper bound, u_i. A value for the lower bound, l_i, can be calculated as follows. Starting from Equation (4), we note that

$$t_i = s_i + \sum M_{ij}\beta_j \prod s_k^{M_{kj}} \tag{10}$$

$$= s_i \left(1 + \sum_{j \in \text{complexes}} M_{ij} \beta_j \prod_{k \in \text{components}} s_k^{M_{kj} - \delta_{ik}} \right)$$

where δ_{ik} is the Kronecker delta.

Rearranging for s_i gives

$$s_i = \frac{t_i}{1 + \sum_{j \in \text{complexes}} M_{ij} \beta_j \prod_{k \in \text{components}} s_k^{M_{kj} - \delta_{ik}}} \tag{11}$$

The lower bound on s_i corresponds to the upper bound on the denominator of Equation (11). For any i, j, and k, either $M_{ij} = 0$, meaning that term does not contribute to the sum in the denominator, or $M_{kj} - \delta_{ik} \geq 0$, meaning every exponent in the denominator is nonnegative. Therefore, since every $s_k \leq u_k$, and each element in the product is either multiplied by zero or raised to a nonnegative exponent, replacing s_k by u_k will increase the value of the denominator, i.e.

$$M_{ij} \beta_j \prod_{k \in \text{components}} s_k^{M_{kj} - \delta_{ik}} \leq M_{ij} \beta_j \prod_{kk \in \text{components}} u_k^{M_{kj} - \delta_{ik}} \tag{12}$$

allowing us to define the lower bound for s_i:

$$\begin{aligned} s_i &\geq \frac{t_i}{1 + \sum_{j \in \text{complexes}} M_{ij} \beta_j \prod_{k \in \text{components}} u_k^{M_{kj} - \delta_{ik}}} \\ &= \frac{t_i u_i}{u_i + \sum_{j \in \text{complexes}} M_{ij} \beta_j \prod_{k \in \text{components}} u_k^{M_{kj}}} \stackrel{\text{def}}{=} l_i \end{aligned} \tag{13}$$

Finally, an initial guess $\boldsymbol{s}_{\text{initial}}$ must be provided as a starting point for the minimisation process. For the first addition of a titration, a simple choice is to use the upper bound, which corresponds to the hypothetical situation where there are no complexes. For each subsequent addition, the initial guess s_i^{initial} can be computed using the total concentration and the optimised value of $\boldsymbol{s}$ from the previous addition, denoted as t_i' and s_i' respectively. This is done by assuming that aliquots of any components added are entirely free, and aliquots of any components removed are removed proportionately from all species that contain that component, as shown in Equation (14).

$$s_i^{\text{initial}} = \begin{cases} s_i' + (t_i - t_i'), & t_i - t_i' \geq 0 \\ s_i' * \dfrac{t_i}{t_i'}, & t_i - t_i' < 0 \end{cases} \tag{14}$$

$\boldsymbol{s}^{\text{initial}}$ is then converted to a corresponding initial guess for $\boldsymbol{x}$ using Equation (7), and the values are clipped to the upper or lower bounds if required.

Polymer speciation

The objective function $G(\boldsymbol{x})$ can be expanded further to account for homopolymers. In the nucleation-growth polymerisation model, two microscopic equilibrium constants are required: the nucleation or dimerisation constant, K_2, and the elongation or growth constant, K_n, in Equation (15) describe the polymerisation of component A.[4]

$$A + A \overset{K_2}{\rightleftharpoons} A_2$$
$$A_2 + A \overset{K_n}{\rightleftharpoons} A_3$$
$$A_3 + A \overset{K_n}{\rightleftharpoons} A_4$$
$$\dots$$
$$A_n + A \overset{K_n}{\rightleftharpoons} A_{n+1} \quad (15)$$

Isodesmic polymerisation is the special case where $K_2 = K_n$. The ratio between K_2 and K_n is often referred to as the interaction parameter or cooperativity factor α,[5] or the nucleation factor σ,[4] which are defined as follows:

$$\alpha = \frac{1}{\sigma} = \frac{K_n}{K_2} \quad (16)$$

Applying Equation (15) more generally to all components in a mixture, we use $\boldsymbol{d}$ for the K_2 of each component, and $\boldsymbol{g}$ for the K_n (setting d_i and g_i to zero if component i does not polymerise). For each component, we can get the expression for the concentration of that component that is part of a homopolymer, p_i:

$$p_i = \sum_{n=2}^{\infty} n[A_n] = \sum_{n=2}^{\infty} n\, d_i\, g_i^{n-2} s_i^n = \frac{s_i^2\, d_i\, (2 - s_i\, g_i)}{(1 - s_i\, g_i)^2} \quad (17)$$

Similarly, we can calculate the total concentration of all homopolymer complexes formed from that component, q_i, by calculating the same sum without the factor of n:

$$q_i = \sum_{n=2}^{\infty} [A_n] = \sum_{n=2}^{\infty} d_i\, g_i^{n-2} s_i^n = \frac{s_i^2\, d_i}{1 - s_i\, g_i} \quad (18)$$

The relationship between the concentrations p_i and q_i is given by

$$\frac{d}{ds_i} q_i = \frac{s_i\, d_i\, (2 - s_i\, g_i)}{(1 - s_i\, g_i)^2} = \frac{p_i}{s_i} \quad (19)$$

In addition, any homopolymer present may form end-capped complexes with one or more of the other components. For example, a component A could form the homopolymer A_n as above, and this polymer could further bind another component X to form the complex X•A_n. By first calculating the concentration of A_n, and then treating this species as a new component, the concentration of X•A_n can be treated as a simple 1:1 complex. If X and A also form the binary complex X•A, this model can be used to describe X acting as an initiator of polymerisation (i.e. if X binds A_n more strongly than it binds A), or as an inhibitor of polymerisation (in the reverse case).

Equation (17) can be expanded to describe end-capped complexes in a straightforward manner. If the equilibrium constant for binding of component j to a homopolymer of component i is given by β, and the stoichiometry of each component in the end-capped complex is given in the vector $\boldsymbol{m}$, then we can obtain the concentrations of the two components in the end-capped polymer:

$$[i \text{ in end-capped polymer}] = \sum^{\infty} \left(n\, d_i\, g_i^{n-2} s_i^n * \beta \quad \prod \quad s_k^{m_k} \right) \quad (20)$$

$$= p_i\beta \prod_{k\in\text{components}} s_k^{m_k}$$

$$[j \text{ in end-capped polymer}] = \sum_{n=2}^{\infty}\left(m_j d_i\, g_i^{n-2} s_i^n * \beta \prod_{k\in\text{components}} s_k^{m_k}\right) \tag{21}$$

$$= m_j q_i \beta \prod_{k\in\text{components}} s_k^{m_k}$$

To include polymers in the set of constraints $\boldsymbol{f}$, each mass balance in Equation (4) must be expanded to also include the concentration of each component that is part of a polymer, or bound to a polymer as an end-cap. To do this, we note that Equations (20) and (21) can be expressed using the partial derivatives of a single function, namely:

$$\frac{\partial}{\partial s_i}\left(q_i\beta \prod_{k\in\text{components}} s_k^{m_k}\right) = \frac{p_i}{s_i}\beta \prod_{k\in\text{components}} s_k^{m_k} = \frac{[i \text{ in end-capped polymer}]}{s_i} \tag{22}$$

$$\frac{\partial}{\partial s_j}\left(q_i\beta \prod_{k\in\text{components}} s_k^{m_k}\right) = \frac{m_j}{s_j} q_i\beta \prod_{k\in\text{components}} s_k^{m_k} = \frac{[j \text{ in end-capped polymer}]}{s_j} \tag{23}$$

Therefore, using $[\boldsymbol{s}\ \boldsymbol{q}]$ to denote the concatenation of the vectors $\boldsymbol{s}$ and $\boldsymbol{q}$ (with $\boldsymbol{q}$ calculated directly from $\boldsymbol{s}$), and expanding the rows of the stoichiometry matrix $\boldsymbol{M}$ to also allow the stoichiometry of a polymer in a complex to be specified, we can rewrite $F(\boldsymbol{s})$ from Equation (6) as Equation (24) which can be minimised as described above.

$$F(\boldsymbol{s}) = \sum_{k\in\text{components}} \left(s_k + q_k - t_k \ln\frac{s_k}{c^{\ominus}}\right) + \sum_{j\in\text{complexes}} \beta_j \prod_{k\in\text{components and polymers}} [\boldsymbol{s}\ \boldsymbol{q}]_k^{M_{kj}} \tag{24}$$

Different boundary conditions must be used for an isotherm that involves polymerisation. For a component i that forms a polymer, $G(\boldsymbol{x})$ is only defined for $s_i < 1/g_i$, as larger values of s_i represent the nonphysical scenario where the concentration of polymer increases indefinitely with increasing length. In this case, t_i cannot be used as the upper bound for s_i. If the component were not part of any equilibrium apart from polymerisation, then the concentration of s_i can be obtained by solving

$$t_i = s_i + p_i = \frac{s_i^2\, d_i\, (2 - s_i\, g_i)}{(1 - s_i\, g_i)^2} \tag{25}$$

Rearranging gives a third-order polynomial in s_i:

$$t_i + (-1 - 2t_i g_i)s_i + \left(-2d_i + 2g_i + t_i g_i^2\right)s_i^2 + \left(d_i g_i - g_i^2\right)s_i^3 = 0 \tag{26}$$

Equation (26) has one real solution in the domain $0 \le s_i \le 1/g_i$. Since any additional equilibria that include component i can only decrease the concentration of s_i, and never increase it, the solution to Equation (26) can be used as the upper bound u_i.

The lower bound l_i also needs to be adapted to include the concentration of the polymers. This can be done in a similar manner to the method described above. Starting from Equation (24) rather than Equation (4), we get

$$\begin{aligned} t_i &= s_i + p_i + \sum_{j \in \text{complexes without } p_\text{i}} M_{ij}\beta_j \prod_{k \in \text{components}} [\boldsymbol{s}\,\boldsymbol{q}]_k^{M_{kj}} \\ &\quad + \sum_{j \in \text{complexes with } p_\text{i}} p_i\beta_j \prod_{k \in \text{components}} [\boldsymbol{s}\,\boldsymbol{q}]_k^{M_{kj}} \\ &= s_i \left(1 + \frac{p_i}{s_i} + \sum_{j \in \text{complexes without } p_\text{i}} M_{ij}\beta_j \prod_{k \in \text{components}} [\boldsymbol{s}\,\boldsymbol{q}]_k^{M_{kj}-\delta_{ik}} \right. \\ &\quad \left. + \sum_{j \in \text{complexes with } p_\text{i}} \frac{p_i}{s_i}\beta_j \prod_{k \in \text{components}} [\boldsymbol{s}\,\boldsymbol{q}]_k^{M_{kj}} \right) \end{aligned} \tag{27}$$

Rearranging for s_i gives

$$\begin{aligned} s_i = t_i \div & \left(1 + \frac{p_i}{s_i} + \sum_{j \in \text{complexes without } p_\text{i}} M_{ij}\beta_j \prod_{k \in \text{components}} [\boldsymbol{s}\,\boldsymbol{q}]_k^{M_{kj}-\delta_{ik}} \right. \\ & \left. + \sum_{j \in \text{complexes with } p_\text{i}} \frac{p_i}{s_i}\beta_j \prod_{k \in \text{components}} [\boldsymbol{s}\,\boldsymbol{q}]_k^{M_{kj}} \right) \end{aligned} \tag{28}$$

The lower bound on s_i corresponds to the upper bound on the denominator of Equation (28), which can be obtained by replacing p_i/s_i with $p_i(u_i)/u_i$, $\boldsymbol{s}$ with $\boldsymbol{u}$, and $\boldsymbol{q}$ with $\boldsymbol{q}(\boldsymbol{u})$, which is defined as the largest possible value of $\boldsymbol{q}$, which it takes when $\boldsymbol{s} = \boldsymbol{u}$. To show that p_i/s_i can be replaced with $p_i(u_i)/u_i$, we note that

$$\frac{p_i}{s_i} = \frac{s_i d_i\,(2 - s_i\,g_i)}{(1 - s_i\,g_i)^2} = \frac{d_i}{g_i} * \frac{1 - (1 - s_i g_i)^2}{(1 - s_i g_i)^2} = \frac{d_i}{g_i}\left(\frac{1}{(1 - s_i g_i)^2} - 1\right) \tag{29}$$

Since $s_i g_i \le u_i g_i < 1$, this means that $p_i/s_i \le p_i(u_i)/u_i$.

Making these substitutions in Equation (28) gives an expression for the lower bound:

$$s_i \geq t_i \div \left(1 + \frac{p_i(u_i)}{u_i} + \sum_{j \in \text{complexes without } p_i} M_{ij}\beta_j \prod_{k \in \text{components}} [\boldsymbol{u}\,\boldsymbol{q}(\boldsymbol{u})]_k^{M_{kj}-\delta_{ik}} + \sum_{j \in \text{complexes with } p_\text{i}} \frac{p_i(u_i)}{u_i}\beta_j \prod_{k \in \text{components}} [\boldsymbol{u}\,\boldsymbol{q}(\boldsymbol{u})]_k^{M_{kj}}\right)$$
$$= t_i u_i \div \left(1 + p_i(u_i) + \sum_{j \in \text{complexes without } p_\text{i}} M_{ij}\beta_j \prod_{k \in \text{components}} [\boldsymbol{u}\,\boldsymbol{q}(\boldsymbol{u})]_k^{M_{kj}} + \sum_{j \in \text{complexes with } p_\text{i}} p_i(u_i)\beta_j \prod_{k \in \text{components}} [\boldsymbol{u}\,\boldsymbol{q}(\boldsymbol{u})]_k^{M_{kj}}\right) \stackrel{\text{def}}{=} l_i \tag{30}$$

The lower bound can be interpreted in terms of the maximum fraction of free *i*:

$$l_i = t_i \frac{\max\,(\text{free } i)}{\max\,(\text{free } i) + \max(i \text{ in polymers}) + \max(i \text{ in complexes})} \tag{31}$$

References

1 F. Gao and L. Han, *Comput. Optim. Appl.*, 2012, **51**, 259–277.

2 P. Virtanen, R. Gommers, T. E. Oliphant, M. Haberland, T. Reddy, D. Cournapeau, E. Burovski, P. Peterson, W. Weckesser, J. Bright, S. J. van der Walt, M. Brett, J. Wilson, K. J. Millman, N. Mayorov, A. R. J. Nelson, E. Jones, R. Kern, E. Larson, C. J. Carey, İ. Polat, Y. Feng, E. W. Moore, J. VanderPlas, D. Laxalde, J. Perktold, R. Cimrman, I. Henriksen, E. A. Quintero, C. R. Harris, A. M. Archibald, A. H. Ribeiro, F. Pedregosa and P. van Mulbregt, *Nat. Methods*, 2020, **17**, 261–272.

3 C. Zhu, R. H. Byrd, P. Lu and J. Nocedal, *ACM Trans. Math. Softw.*, 1997, **23**, 550–560.

4 D. Zhao and J. S. Moore, *Org. Biomol. Chem.*, 2003, **1**, 3471–3491.

5 C. A. Hunter and H. L. Anderson, *Angew. Chem. Int. Ed.*, 2009, **48**, 7488–7499.
